# Inhibitory Effect of Punicalagin on Inflammatory and Angiogenic Activation of Human Umbilical Vein Endothelial Cells

**DOI:** 10.3389/fphar.2021.727920

**Published:** 2021-11-16

**Authors:** Wei Liu, Yanghui Ou, Yumeng Yang, Xuemei Zhang, Liqi Huang, Xiaohua Wang, Buling Wu, Mingcheng Huang

**Affiliations:** ^1^ Shenzhen Stomatology Hospital (Pingshan) of Southern Medical University, Shenzhen, China; ^2^ Department of Digestive Medicine Center, The Seventh Affiliated Hospital, Sun Yat-Sen University, Shenzhen, China; ^3^ School of Stomatology, Southern Medical University, Guangzhou, China; ^4^ Department of Nephrology, Center of Nephrology and Urology, The Seventh Affiliated Hospital, Sun Yat-Sen University, Shenzhen, China

**Keywords:** punicalagin, inflammation, endothelial cells, angiogenesis, NF-κB

## Abstract

Punicalagin, a major ellagitannin isolated from pomegranate, is proved to have various pharmacological activities with an undefined therapy mechanism. The objective of this research was to demonstrate the effect of punicalagin on anti-inflammatory and angiogenic activation in human umbilical vein endothelial cells (HUVECs) and their potential mechanisms. Endothelial-leukocyte adhesion assay was applied to evaluate primary cultures of HUVECs activation following tumor necrosis factor alpha (TNF-α) treatment. The endothelial cell proliferation, migration, permeability and tube formation were assessed by EdU assay, wound migration assay, trans-endothelial electrical resistances (TEER) assay, and capillary-like tube formation assay, respectively. In addition, the expression of relevant proteins was assessed using Western blot analysis. We confirmed that punicalagin could reduce the adhesion of human monocyte cells to HUVECs *in vitro* and *in vivo*. Further, punicalagin decreased the expression of mRNA and proteins of ICAM-1 and VCAM-1 in HUVECs. Moreover, punicalagin inhibited permeability, proliferation, migration, and tube formation in VEGF-induced HUVECs, suppressed IKK-mediated activation of NF-κB signaling in TNF-α-induced endothelial cells, and inhibited vascular endothelial growth factor receptor 2 (VEGFR2) activation and downstream p-PAK1. Our findings indicated that punicalagin might have a protective effect on HUVECs activation, which suggested that punicalagin functions through an endothelial mediated mechanism for treating various disorders such as, cancer, rheumatoid arthritis, and cardiovascular disease.

## Introduction

Vascular endothelium separates blood flow from the surrounding tissues. The dysfunction of vascular endothelium is closely correlated with a variety of disorders, including but not least tumor, rheumatoid arthritis, atherosclerosis, sepsis and inflammatory bowel diseases (Pober and Sessa, 2007; Roifman et al., 2009; Khan et al., 2010; Sun et al., 2019). Moreover, leukocyte transport through endothelium to sites of inflammation is regulated by multi-stage processes. It has been revealed that the expression of vascular cell adhesion molecule 1 (VCAM-1) and intercellular adhesion molecule 1 (ICAM-1) were upregulated in vascular endothelium under inflammatory conditions, which is essential for mediating early leukocyte attachment and related events (Huang et al., 2017; Li et al., 2018). Furthermore, increased vascular permeability is also critical for tissue inflammation and angiogenesis. Loosening of established endothelial cell-cell junctions is required for recruitment of leukocytes from blood into the subendothelial space and for new capillaries to develop from pre-existing vessels (Cirino G et al., 2003). However, the precise mechanisms underlying these processes remain unclear.

Several signaling pathways have been found to be pivotal players in regulating endothelial inflammation, permeability and angiogenesis. In the vascular endothelium, increased nuclear factor-κB **(**NF-κB) activity resulted in the expression of inflammation-related proteins, including those encoding proinflammatory cytokines, chemoattractant proteins and adhesion molecules (Sun, 2017; Mussbacher et al., 2019; Pflug and Sitcheran, 2020). Targeting NF-κB-mediated activation of endothelial cells would offer an innovative approach in anti-inflammatory treatments. Additionally, p21-activated kinases (PAKs), a family of serine/threonine kinases, are critical effector proteins of Rac1, Rho GTPases, and Cdc 42 (Rane and Minden, 2014; Bu et al., 2019). They may be subdivided into PAK1-3 and PAK4-6 in terms of their structural organization and mode of regulation (Khan et al., 2020). They undergo auto-phosphorylation at multiple sites (Martin et al., 1995; Manser et al., 1997) and have been found to be essential for regulating cell motility, transcription, death and survival (Kumar et al., 2006). It has been proved that normal PAK1 activity is indispensable to endothelial motility and permeability (Lv et al., 2018; Wu et al., 2021) and that its inhibition may suppress angiogenesis (Khan et al., 2020).

Punicalagin is an abundant ellagic acid which may be extracted from pomegranate peel. It has been widely applied in traditional Chinese medicine, functional foods, and cosmetics (Xu et al., 2017). Numerous researches have revealed that punicalagin has significant effects against inflammation, oxidation, cancer, fungi, and bacteria (Xu et al., 2014; Zou et al., 2014; Cao et al., 2015; Kumagai et al., 2015; Xu et al., 2015; Wang et al., 2016). It has been found to inhibit the production of lipopolysaccharide-induced inflammatory mediators in RAW264.7 macrophages by controlling the activation of mitogen-activated protein kinase and NF-κB (Xu et al., 2014). It was also found that punicalagin to attenuated the neuroinflammatory response of primary rat microglia activated by lipopolysaccharide (Olajide et al., 2014). These findings indicate the role for punicalagin to regulate inflammatory responses, but its effect on the vascular endothelium remains unknown. Therefore, this research aimed to investigate the potential role of punicalagin in modulating endothelial permeability, inflammation, angiogenesis, and the related underlying mechanisms.

## Materials and Methods

### Reagents and Materials

Punicalagin (purity >98%) was obtained from Cayman Chemical (Ann Arbor, United States) and dissolved in dimethyl sulfoxide (DMSO). Human recombinant tumor necrosis factor alpha (TNF-α), VEGF165, and ELISA kits for interleukin-6 (IL-6), interleukin-8 (IL-8), ICAM-1, VCAM-1 and monocyte chemotactic protein 1 (MCP-1) were obtained from Cell Signaling Technology (Danvers, MA, United States). Collagenase II, DMEM and Penicillin-Streptomycin Mixed Solution were purchased from Thermo Fisher Scientific (Gibco, Grand Island, United States). Antibiotics, trypsin/EDTA, and phosphate buffered solution (PBS) were purchased from Solarbio (Beijing, China). EBM-2 and EGM-2 media were obtained from LONZA (United States). Antibodies against VCAM1, ICAM1, IKBα, phospho-IKBα, phospho-VEGFR2, VEGFR2, phospho-PAK1, PAK1 and NF-κBp65 were obtained from Santa Cruz Biotechnology. Sterile plastic material was obtained from Corning Inc. (NY, United States). Fetal bovine serum (FBS), reagents and materials for Western Blot analysis were purchased from Sigma-Aldrich (St. Louis, MO, United States).

### Isolation and Culture of HUVECs

The research protocol was approved by the Seventh Affiliated Hospital, Sun Yat-sen University Research Ethics Committee. This study was carried out according to the Declaration of Helsinki. After informed consent, HUVECs were obtained by enzymatic digestion after separating the tissue from the fresh umbilical cords (Jaffe et al., 1973). The cells were grown in EGM-2 medium, supplemented with 10% FBS and other essential factors. After that, cells were grown in the cell incubator containing 5% carbon dioxide at 37°C, and the culture medium was changed every 3 days. The cells from 4 to 6 passage were utilized for experiments. Before the start of the experiment, HUVECs were starved with serum-free medium.

### Cell Viability Assay

When the cells grow to 80–90% confluence in the petri dish, they were digested with trypsin-EDTA solution-0.25% (Sigma-Aldrich, St. Louis, MO, United States), harvested and serially passaged. HUVECs were then seeded into a 96-well plate at 5×10^4^ cells/ml until they reached 80% confluence and cultured in a serum-free medium overnight. After that, they were exposed to DMSO or different concentrations of punicalagin (12.5, 25, and 50 μM) for 48 h. Subsequently, 10 μL MTT (Beyotime Biotech, Shanghai, China) solution was added to plate and continued to incubate for 4 h. Adding 100 μl of Formazan solution to each well until it is completely dissolved and then measuring the absorbance at 570 nm in a microplate reader (PerkinElmer, Shanghai, China).

### RNA Extraction, qRT-PCR Analysis

After pretreatment with punicalagin for 48 h and exposure to TNF-α for 8 h, total RNA of endothelial cells (ECs) was collected using TRIzol (Invitrogen, Life Technologies NY, United States). cDNA was synthesized with Reverse Transcription Kit (EZBioscience, Roseville, United States). Quantitative real time polymerase chain reaction (qRT-PCR) analysis for the expression of VCAM-1, ICAM-1, IL-6, IL-8 and MCP-1 was carried out with the QuantiTect SYBR Green RT-PCR Kit on Roche LightCycler®480 (Roche, Indianapolis, IN, United States). Relative mRNA expression was normalized by glyceraldehyde-3-phosphate dehydrogenase (GAPDH) expression. Fold change values were calculated using 2^−ΔΔCt^ method. The primers of the target gene are listed in Supplementary Table S1.

### Endothelial-Monocytic Cell Adhesion Assay

To assess the inhibitory effect of punicalagin on the adhesion of white blood cells to ECs, Cell Biolab’s CytoSelect™ Leukocyte-endothelium Adhesion Assay was performed. HUVECs were maintained in Fibrinogen-coated 48-well plates at 80–90% cell confluence and pretreated with DMSO (control group) or punicalagin (12.5, 25, and 50 μM) for 24 h. The samples were then incubated with TNF-α for 4 h. After the above procedures, LeukoTracker™ solution-loaded leukocytes were added to each well. Nonadherent cells were removed after incubation for 1 h. After aspirating the final wash media, Lysis Buffer was added to the plate. The adhesion of PBMC in each group was observed with a microscope (Leica, Germany). At least three random fields of adherent leukocytes were counted per well. At the same time, the adherent cells in each well were counted three times, and the average value was taken.

### Transwell Assay for Vertical Trans-Migration of Peripheral Blood Mononuclear Cell

The Boyden chamber assay (Transwell, Corning Inc., United States) was used to determined migration ability of PBMC. In short, HUVECs were resuspended in EGM-2 medium and added to each lower chamber. When the cell growth reached 90% confluence, HUVECs were cultured in basal media for 12 h. The cells were incubated with punicalagin at concentrations of 12.5, 25, 50 μM, or DMSO for 24 h. The samples were then treated with human recombinant TNF-α for 4 h. After injecting human PBMC into the upper chamber, they were incubated for 2 h, and then the noninvading cells were removed. Finally, the PBMC that migrated from the upper chamber were collected, treated with methanol and stained with 4% crystal violet for 15 min, and counted under a microscope and calculated the relative amount. Data are expressed as mean ± standard.

### ELISA Assay

The cells were pretreated with TNF-α (10 ng/ml) with or without punicalagin (12.5, 25, and 50 μM). The supernatant of each group of cells was collected after 24 h.The concentration of pro-inflammatory cytokines and adhesion molecules in cell culture supernatant were analyzed by ELISA assay (R&D Systems, Minneapolis, United States). The experimental procedures strictly followed manufacturer’s instructions. All tests were performed in triplicate.

### Measurement of TEER

HUVECs were seeded on the polycarbonate membrane of a 24-well Transwell culture plate at 3 × 10^4^cells/ml. When the cells were cultured to 80% confluence, HUVECs treated with medium containing punicalagin (12.5, 25, 50 μM) or DMSO for 12 h. Replace vascular endothelial growth factor (VEGF) or PBS for 4 or 16 h. The resistance was detected using the cell resistance meter (Millicell-ERS; Millipore, Billerica, MA, United States). The two stages of the electrode are inserted into the solution respectively, and the resistance value is read after the value on the resistance meter is stable. Finally, the TEER value is obtained by calculation.

### Tube Formation Assay

First, the cells were pretreated with different concentrations of punicalagin. The 24-well plate and Matrigel (Geltrex; Thermo Fisher Scientific, MA, United States) were pre-cooled in advance. Matrigel was added to the well to make it solidify at 37°C. And then, the pretreated HUVECs were seeded. The tubules were observed and photographed with fluorescence microscopy after 8 h. Quantitative analysis of tubules was performed using AngioSys 2.0 image analysis software (Cellworks, Buckingham, United Kingdom). This assay was replicated three times.

### EdU Proliferation Assay

HUVECs were seeded onto 24-well plates at a density of 1 × 10^5^cells/ml. After the cells adhere to the well, punicalagin was added for pretreatment for 24 h. And then, medium was changed with the prepared 50 μM EdU (Ribobio, Guangzhou, China) medium and incubated cells for 2 h. The cells were then fixed with methanol, stained with fluorescent dyes and washed several times. Finally, the positive cells were observed and counted with fluorescence microscopy (Olympus, Tokyo, Japan).

### Wound Healing Assay for Horizontal Migration

The punicalagin was detected for its effect on the horizontal migration of HUVECs by scratch wound healing assay. In brief, HUVECs were seeded into 6-well plates, when they were grown at 37°C until 80% confluence, treated with punicalagin at different concentrations (12.5, 25, 50 μM) for 12 h, followed by stimulation with 10 ng/ml TNF-α. The artificial wounds were scratched on the monolayer using a 1-ml micropipette tip. And then, the debris were washed with PBS, added serum-free medium 1.5 ml/per well. After 24 h, the cells in the wound area were evaluated and the images of cells across the scratch were captured under the brightfield microscope. Software of Image 1.47 was used to analyze the number of migrated cells.

### Immunofluorescence Staining

HUVECs were seeded in cell slides, and when they were fused to 90–95%, they were pretreated with puncalagin or DMSO for 12 h, and then stimulated with TNF-α or PBS for 30 min. Next, they were washed 3 times with cold PBS, 10 min each time. HUVECs were subsequently fixed with methanol, permeabilized with 0.2% cold TritonX-100. Samples were incubated with primary antibody (anti-p65) overnight at 4°C. Then washed with cold PBS and blocked with 5% BSA. Finally, HUVECs were incubated with DAPI for nuclear staining. Samples were observed with the confocal fluorescence microscopy Zeiss LSM710 (Carl Zeiss, Oberkochen, Göttingen, Germany).

### Western Blot Analysis

After treatment with or without punicalagin, the cells were stimulated with TNF-α or VEGF for 15 min. ICAM1, VCAM1, Phosphorylation of IκBα, IκBα, phosphorylation of VEGFR2,VEGFR2, PAK1 and phosphorylation of PAK1 were analyzed using western blot analysis. HUVECs were lysed by RIPA buffer to obtain the total protein from the cells on ice, after which the mixture was centrifuged at 15,000 rpm, 4°C for 15 min to separate cell debris. Transfer supernatants to new tubes for further analysis. Next, adding loading buffer to the supernatants and heating at 95°C for 5 min to denature the protein. All equivalent amounts of samples were resolved in 10% SDS-PAGE gels and then transferred to PVDF membranes, which were immunoblotted with the specified antibodies overnight at 4°C, such as ICAM1, VCAM1, anti-phospho-VEGFR2, VEGFR2, anti-phosphor-IκBα, anti-phosphor-IκB kinase (IKK), anti-IκBα, PAK1 and anti-phosphor- PAK1. Then, the membranes were incubated with the corresponding secondary antibody (1:2,000, Santa Cruz Biotechnology) at 37°C for 1 h. At last, the antibody-antigen complexes were visualized with the Western Blot Imaging System. The gray value of protein bands quantification was done by the software program ImageJ. Levels of protein were normalized GAPDH.

### Model of Acute Lung Inflammation

The animal study was conducted in accordance with guidelines from Directive 2010/63/EU of the European Parliament on the protection of animals for scientific purposes. The animal experiment protocol was approved by the Animal Care and Ethics Committee, Sun Yat-sen University. Thirty male C57BL/6J mice were randomly divided into three groups (*n* = 10 per group): 1) control group; 2) TNF-α group; 3) TNF-α+punicalagin group. Before inducing acute pneumonia, punicalagin (50 mg/kg/d) was administered by intraperitoneal injection on each of 5 days. On the following day, 2 μg TNF-α was intraperitoneally injected to each mouse to induce lung injury. 4 h later, all animals were euthanized by overdose of pentobarbital delivered intraperitoneally, so that tissues and organs could be harvested for analysis. H&E staining were performed on lung tissue. Myeloperoxidase (MPO) activity was measured in lung homogenates by the Amplex Red Peroxidase Assay Kit (Invitrogen, Carlsbad, CA, United States) according to the manufacturer’s instructions. The content of IL-6 was detected by Elisa. Lung tissues were harvested and frozen in liquid nitrogen immediately until homogenization. Proteins were extracted from the lungs and detected by Western blot.

### Statistical Analysis

Each experiment was performed in triplicate. The values were expressed as mean ± standard deviation (SD). Student’s t-test or one-way analysis of variance was used to compare the statistical significance of the differences. Data analyses were performed by using Statistical Package for Social Sciences (SPSS) version 23 database (SPSS Inc., Chicago, IL, United States). Level of significance was defined as 0.05. The figures were constructed with GraphPad Prism8 (Graph-Pad Software, San Diego, CA, United States).

## Results

### Effects of Punicalagin on HUVECs Viability

**FIGURE 1:**
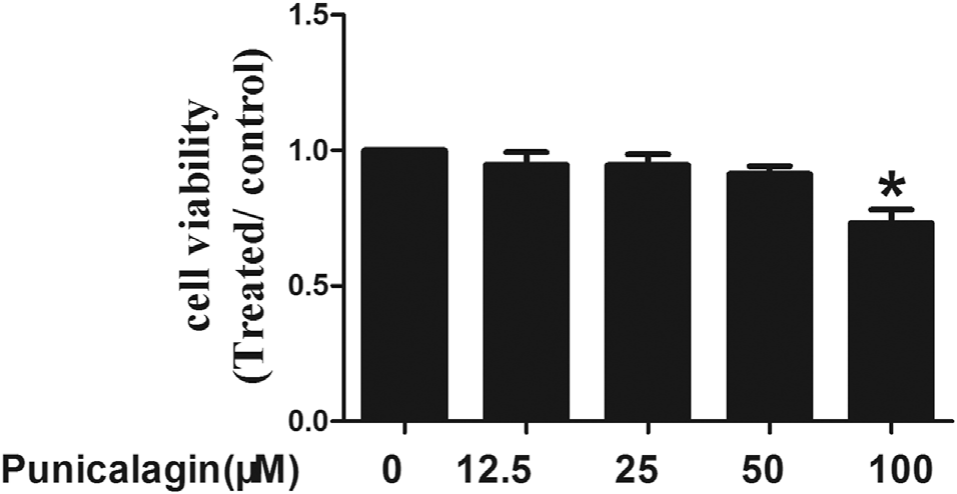
Effects of punicalagin on HUVECs viability. HUVECs were stimulated with punicalagin for 48 h at various concentrations. The control group was treated with DMSO. Cell viability was determined using MTT assay. This result was repeated in triplicate. *p < 0.05 vs. control group.

The effect of punicalagin concentration on HUVECs was determined by MTT. We found that compared with the control group, punicalagin at low concentrations (12.5, 25 and 50 μM) showed no effects on HUVECs. However, the viability of HUVECs decreased significantly by punicalagin treatment at the concentration of 100 μM (*p* < 0.05) (Figure 1).

### Inhibition of Punicalagin on Adhesion Molecule in TNF-α-Simulated HUVECs

**FIGURE 2:**
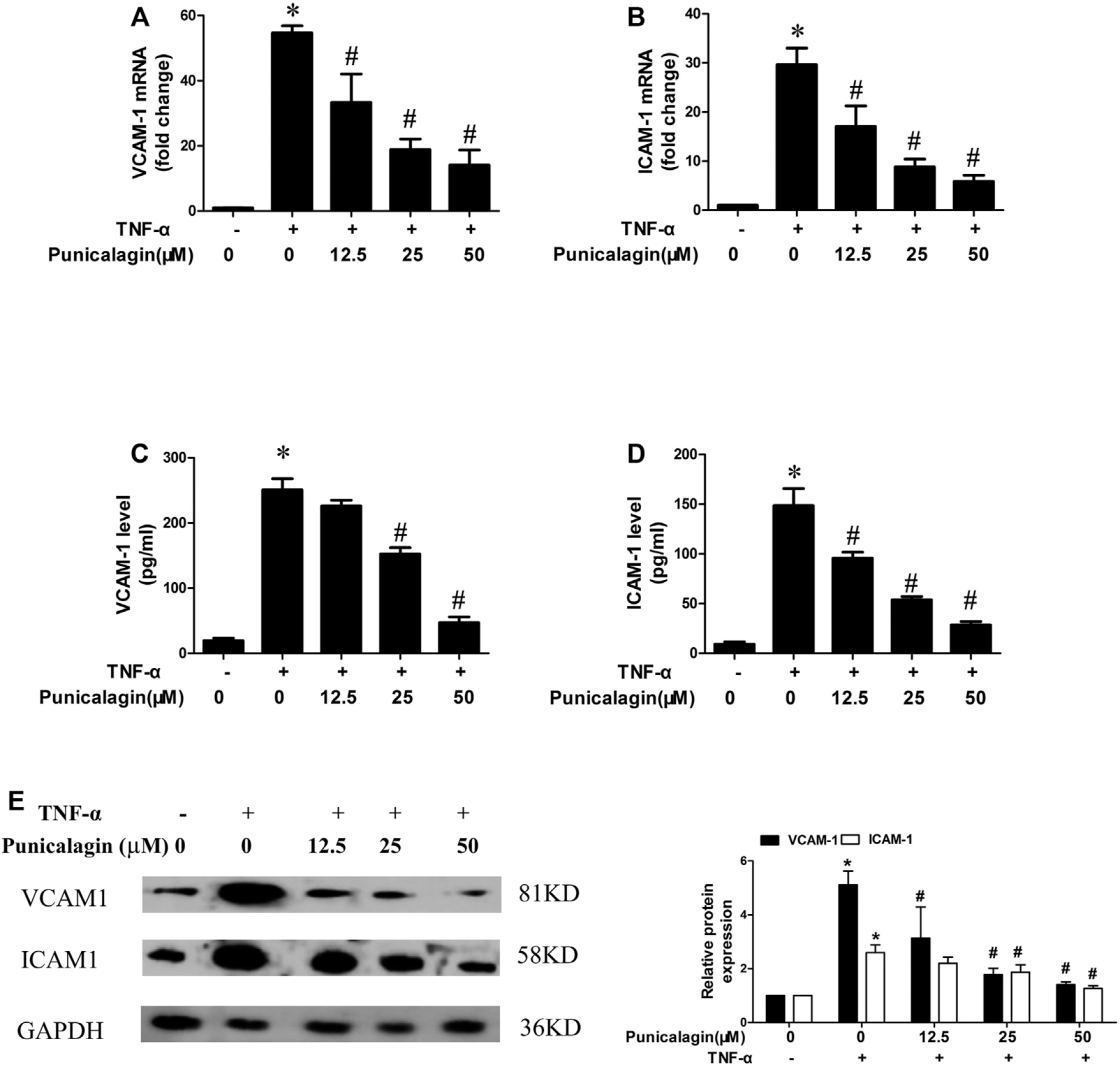
Effect of punicalagin on expressions of VCAM-1 and ICAM-1 in TNF-α-stimulated HUVECs. HUVECs were pretreated with punicalagin and stimulated by TNF-α (10 ng/ml) for 24 h **(A, B)** Gene expression of VCAM-1 and ICAM-1 in HUVECs determined quantitatively using qRT-PCR. **(C, D)** The secreted protein of VCAM-1 and ICAM-1 in the supernatant of each group was detected by ELISA. Western blot analysis of VCAM-1 and ICAM-1 in HUVECs treated with or without punicalagin after treatment with or without 10 ng/ml TNF-α for 8 h **(E)**. The results were performed in triplicate. *p < 0.05 vs. HUVECs without TNF-α, ^#^ *p* < 0.05 vs treatment with TNF-α alone.

Adhesion molecules including VCAM-1, ICAM-1 induce leukocytes adhesion to the endothelial cells (Yang et al., 2016). qRT-PCR was performed to detect the expression of ICAM-1 and VCAM-1 mRNA in the cells, and the supernatant was collected for ELISA. As shown in Figures 2A,B, HUVECs expressed more adhesion molecules in the inflammatory environment, and the pretreatment of punicalagin significantly reduced their expression in a concentration-dependent manner. Similarly, the ELISA results showed that punicalagin treatment reduced the secreted of VCAM-1 and ICAM-1 in the supernatant (Figures 2C,D) (*p* < 0.05). As shown in Figure 2E, punicalagin treatment also suppressed TNF-α-induced VCAM-1 and ICAM-1 protein expression in HUVEC.

### Suppression of Punicalagin on Adhesion of PBMC to TNF-α-Stimulated HUVECs

**FIGURE 3:**
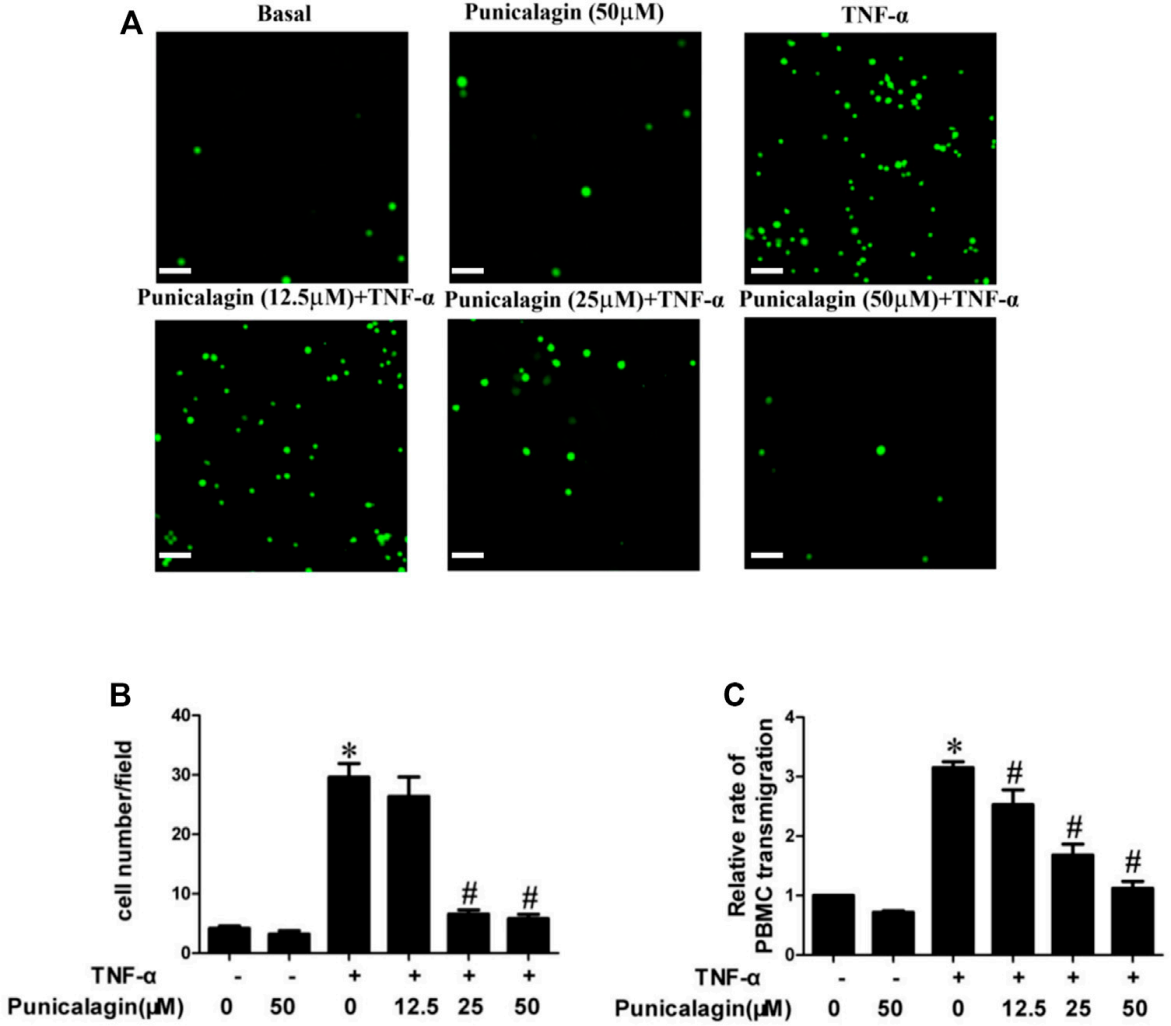
Inhibition of punicalagin on adhesion of PBMC to ECs in inflammation. **(A, B)** Figure A shows the adhesion of PBMC observed in different culture conditions under an inverted fluorescence microscope. **(B)** The quantity of adherent cells. **(C)** Results of Transwell migration assay to explore the effect of punicalagin on TNF-α-induced leukocyte migration across the endothelium. *p < 0.05 vs HUVECs without TNF-α, ^#^ *p* < 0.05 vs treatment with TNF-α alone.

To further prove the possible role of punicalagin in endothelial activation, we observed and calculated the number of PBMC to HUVECs with the fluorescence microscope. Figures 3A,B showed that treatment with punicalagin alone did not affect PBMC’s adherence to HUVECs while TNF-α stimulation significantly increased adhesion of PBMC to HUVECs (*p* < 0.05). However, the adhesion capability was significantly blocked by pretreatment with punicalagin. Besides, we also found that TNF-α significantly induced the transendothelial migration of PBMC, and that this effect was reversed by pretreatment with punicalagin (Figure 3C).

### Inhibitory Effect of Punicalagin on Expression of Pro-inflammatory Factors in HUVECs

**FIGURE 4:**
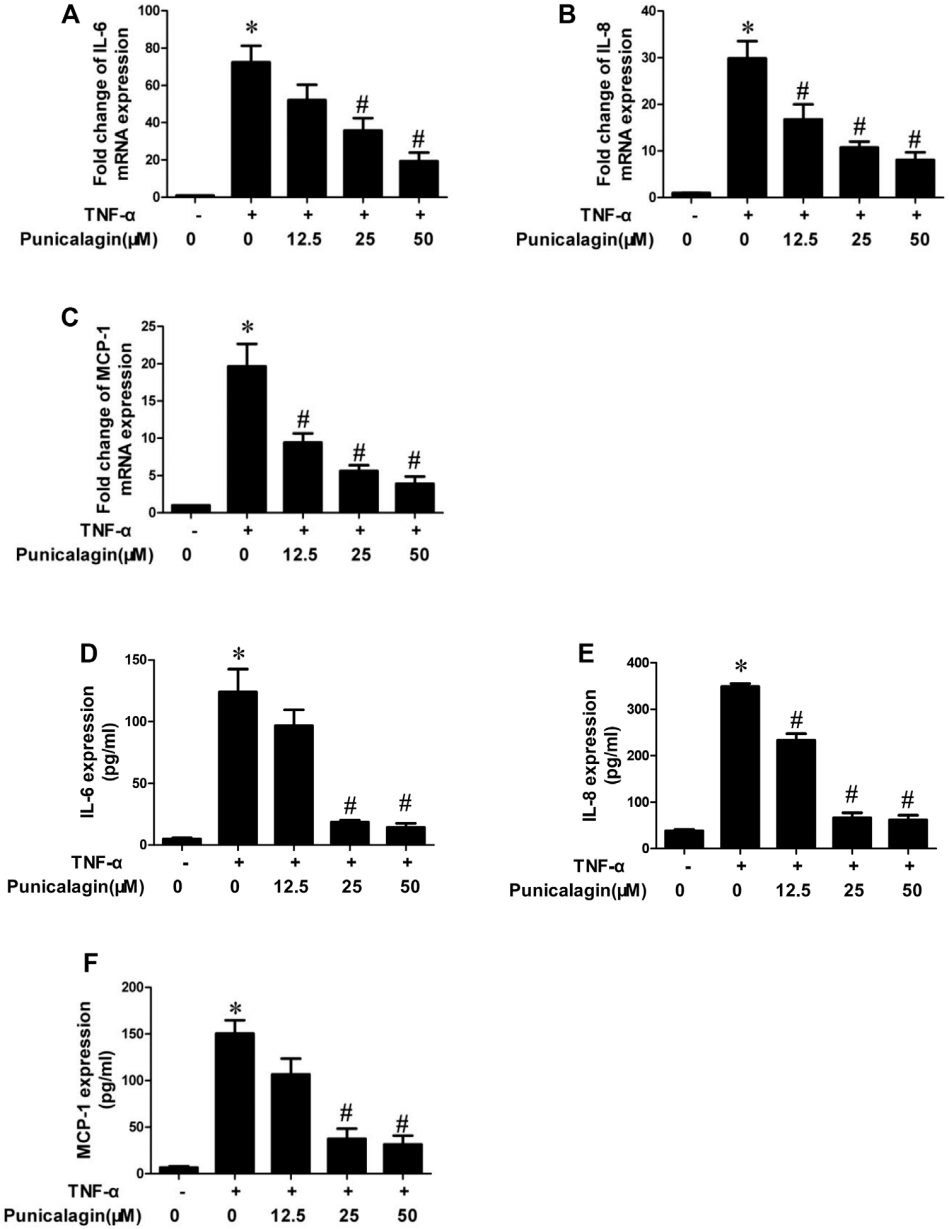
Inhibition of punicalagin on production of TNF-α-induced pro-inflammatory cytokines in HUVECs. **(A–C)** mRNA expression levels of pathogenic factors related to inflammation in cells: IL-6, IL-8 and MCP-1. **(D–F)** Protein content in cell supernatant was detected by ELISA. *p < 0.05 vs. HUVECs without TNF-α, ^#^ *p* < 0.05 vs. groups treated with TNF-α alone.

To further determine whether punicalagin has the potential to protect endothelial cells from inflammatory damage, we also tested inflammatory factors from both gene expression and quantification. As shown in Figures 4A–C, the qRT-PCR analysis showed that pretreatment with punicalagin significantly attenuated TNF-α-stimulated mRNA expression of IL-6, IL-8, and MCP-1 in HUVECs (*p* < 0.05). Similarly, ELISA results showed that punicalagin significantly could rescue the abnormal elevation of IL-6, IL-8, and MCP-1 induced by TNF-α in the culture medium (*p* < 0.05) (Figures 4D–F).

### Suppression of Punicalagin on VEGF- Induced Endothelial Permeability

**FIGURE 5:**
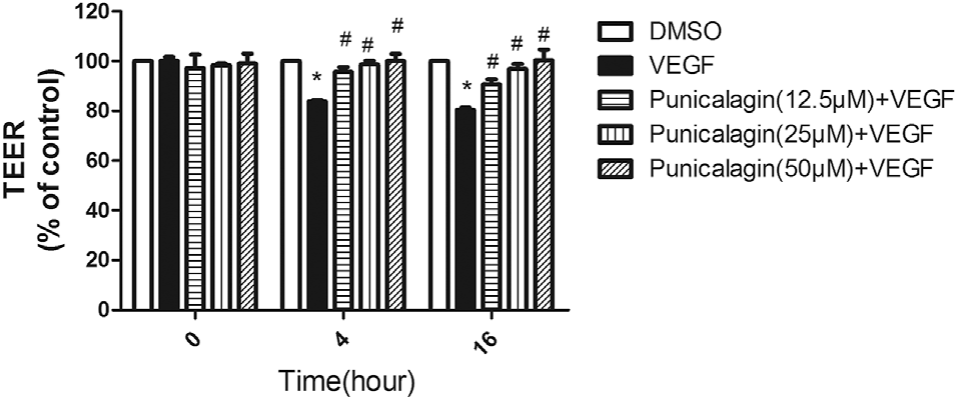
Effect of punicalagin on vascular hyperpermeability *in vitro*. Electrical resistance of a monolayer of HUVECs was measured by transendothelial electrical resistance (TEER). The experiments were performed in triplicate. Data are represented ad mean ± SD. *p < 0.05 vs. DMSO groups, ^#^ *p* < 0.05 vs. treatment with VEGF alone.

To evaluate the involvement of punicalagin in VEGF-induced endothelial barrier dysfunction, we used *in vitro* measurement of the changes in transendothelial resistance (TEER). As shown in Figure 5, pretreatment with punicalagin for 4 and 16 h significantly attenuated VEGF-induced reduction in TEER in HUVECs (*p* < 0.05).

### Suppression of Punicalagin on Neovascularization

**FIGURE 6:**
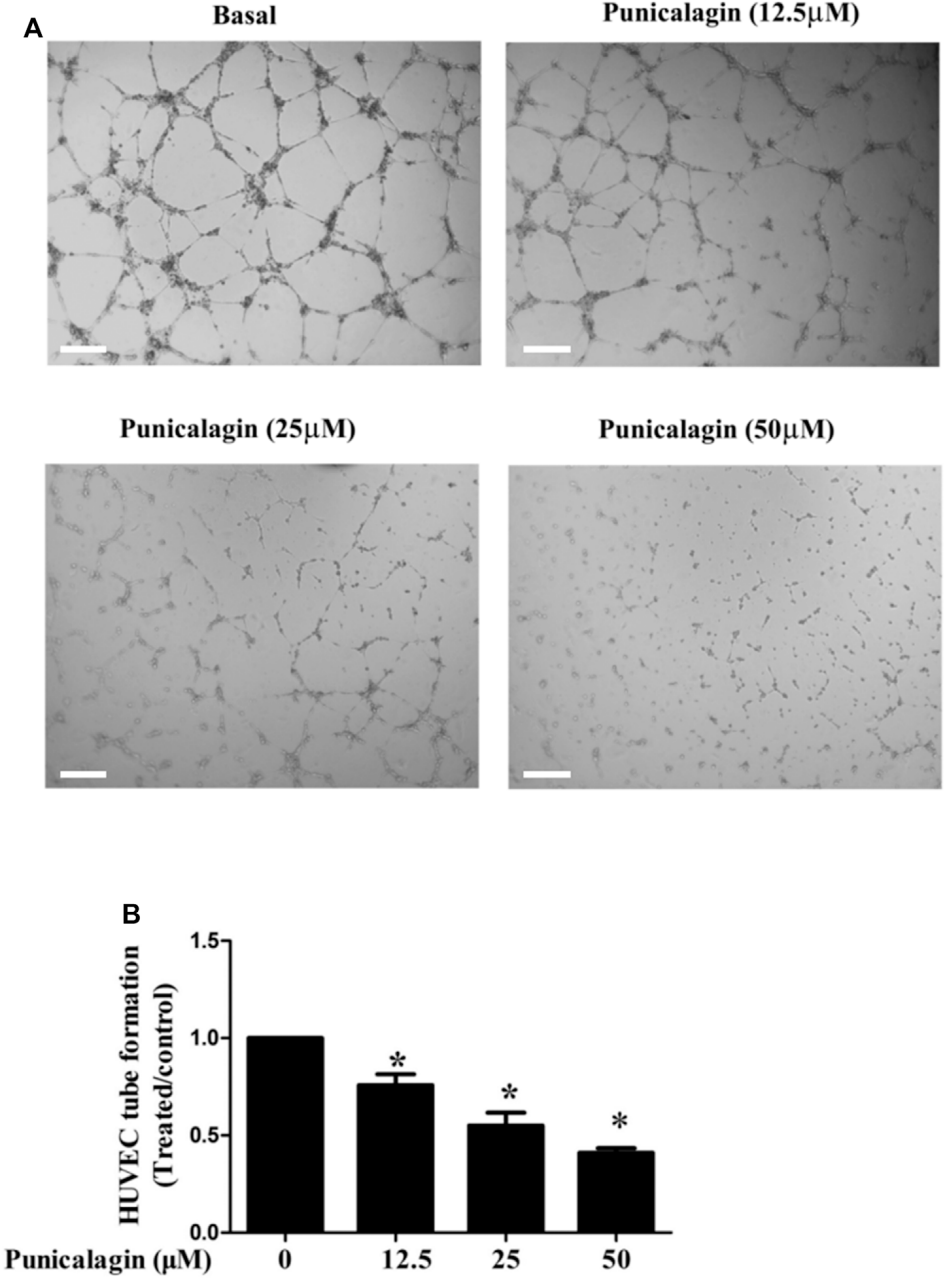
Effects of punicalagin on HUVECs tube formation. **(A)** The effect of punicalagin on the formation of tubular structure in endothelial cells. HUVECs were seeded on Matrigel layer, after they were resuspended with punicalagin containing 12.5, 25, and 50 μM. The formation of vascular-like structures in different concentration groups after 8 h was evaluated. **(B)** The histogram represents the dose response for punicalagin in tube formation of HUVECs. *p < 0.05, punicalagin treatment vs. control group.

**FIGURE 7:**
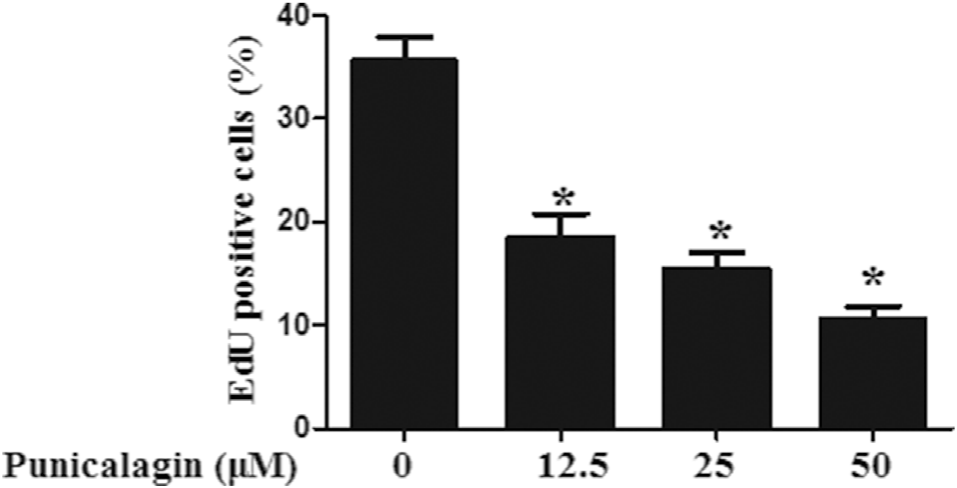
Inhibitory effect of punicalagin on proliferation of HUVECs. The histogram represents the percentage of positive cells in the total cells under the microscope. Values are represented as mean ± SD. *p < 0.05, punicalagin treatment vs. control group.

**FIGURE 8:**
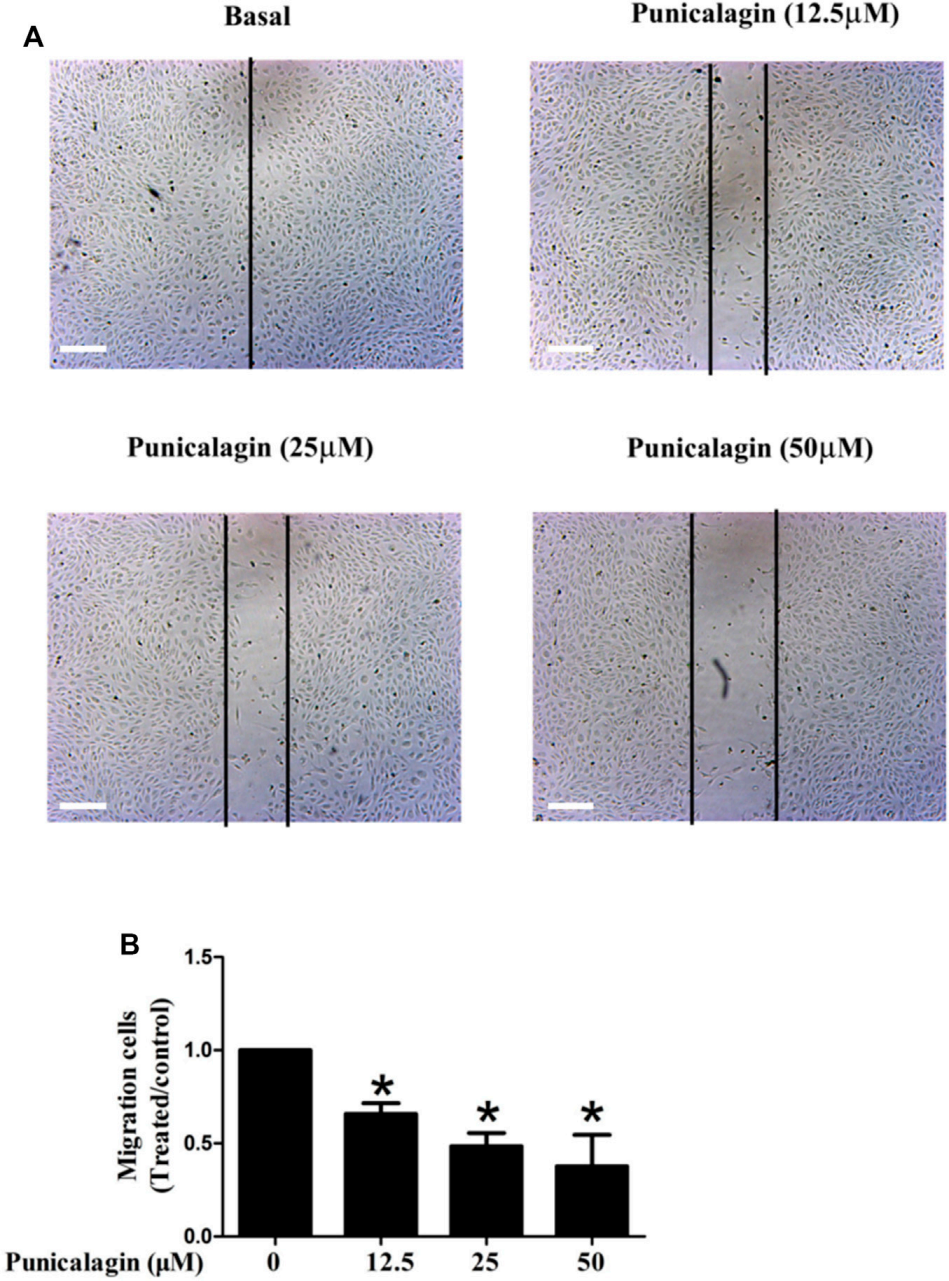
Effects of punicalagin on HUVECs migration **(A, B)**. Inhibition of punicalagin on HUVECs migration in wound healing assay (original magnification, ×50). *p < 0.05, punicalagin treatment vs. control group.

The anti-angiogenesis ability of punicalagin was assessed by tube formation assay *in vitro*. First, we explored the effect of punicalagin on the formation of blood vessels in HUVECs. It was found that pretreatment of cells with punicalagin inhibit the formation of blood vessel-like structures (Figure 6). In the process of angiogenesis, the proliferation and migration of endothelial cells play the key step. Therefore, to detect the effect of punicalagin on cell proliferation, we performed the EdU cell proliferation assay. As shown in Figure 7, punicalagin significantly suppressed the proliferation of HUVECs (*p* < 0.05). Endothelial cell migration is an important process in angiogenesis and functionally differs from proliferation. The effects of punicalagin on the chemotactic motility of HUVECs were assessed by wound-healing migration. EBM-2 with multiple growth factors triggered cell motility, but this effect was dose-dependently inhibited by punicalagin (Figure 8).

### Inhibition of Punicalagin on Activation of NF-κB Signaling Pathway

**FIGURE 9:**
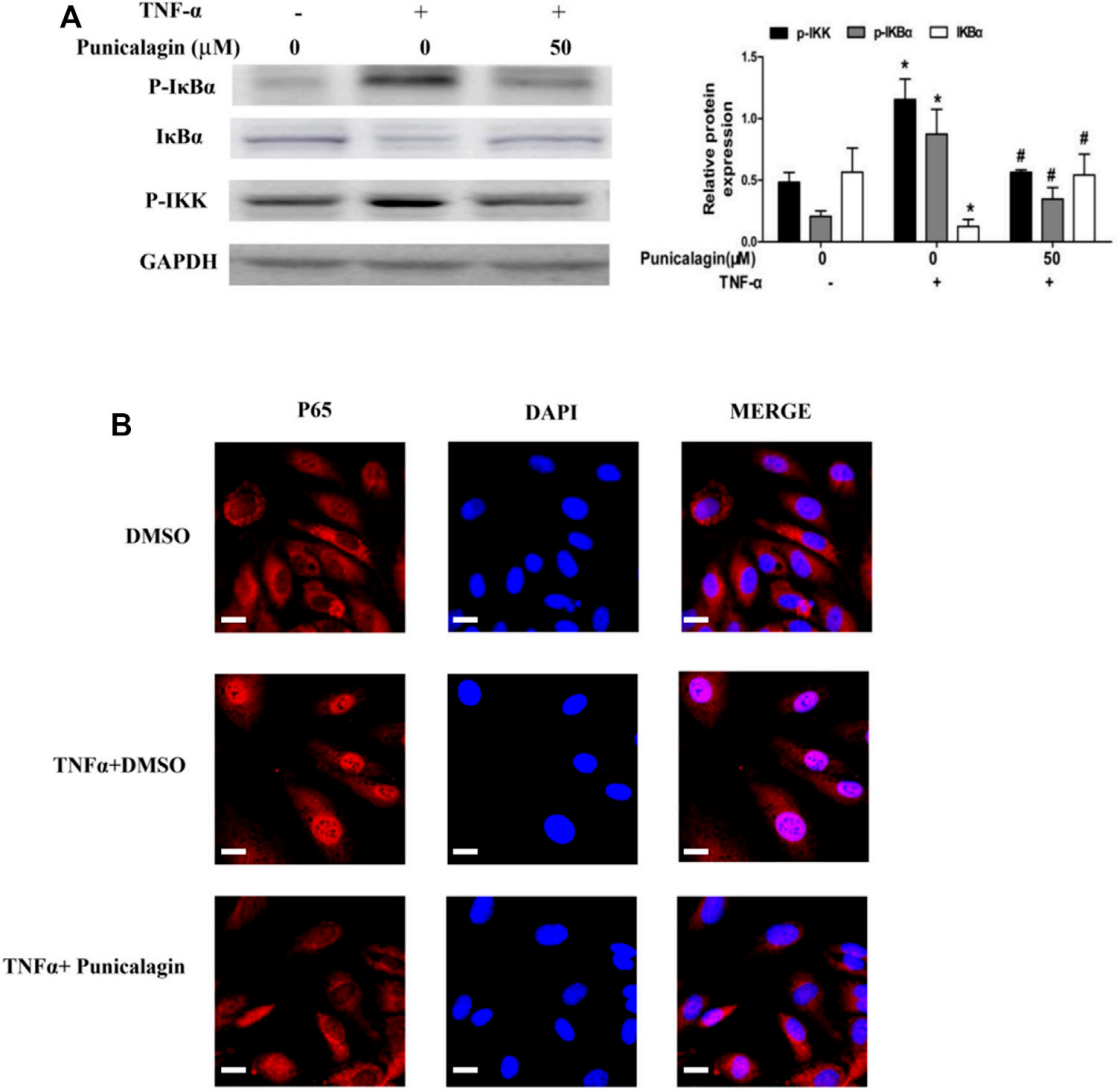
Regulatory effect of punicalagin on NF-κB signaling pathway of HUVECs. **(A)** Difference in the expression of phosphorylated IκBα, phosphorylated IKK and IκBα with DMSO (control) and punicalagin (50 µM). **(B)** Position of p65 in cells tracked by immunofluorescence staining. Data are represented as mean ± SD. *p < 0.05, vs. HUVECs without TNF-α, ^#^ *p* < 0.05 vs. groups treatment with TNF-α alone.

To explore the anti-inflammatory mechanism of punicalagin, we assessed NF-κB activation and nuclear translocation of the cells by western blot analysis and immunofluorescence staining. In the study, we found that punicalagin (50 μM) decreased phosphorylated IKKβ in TNFα-stimulated ECs. Punicalagin treatments also inhibited the phosphorylating and degradation of IκBα caused by TNF-α stimulation(Figure 9A). (*p* < 0.05). Immunofluorescence staining analysis suggested that the amount of p65 in the nucleus increased with TNF-α stimulation. Pretreatment with punicalagin (50 µM) suppressed the translocation of p65 translocated to the nucleu from cytoplasm, as compared with TNF-α treatment alone (Figure 9B).

### Inhibition of Punicalagin on VEGFR2 Phosphorylation in HUVECs

**FIGURE 10:**
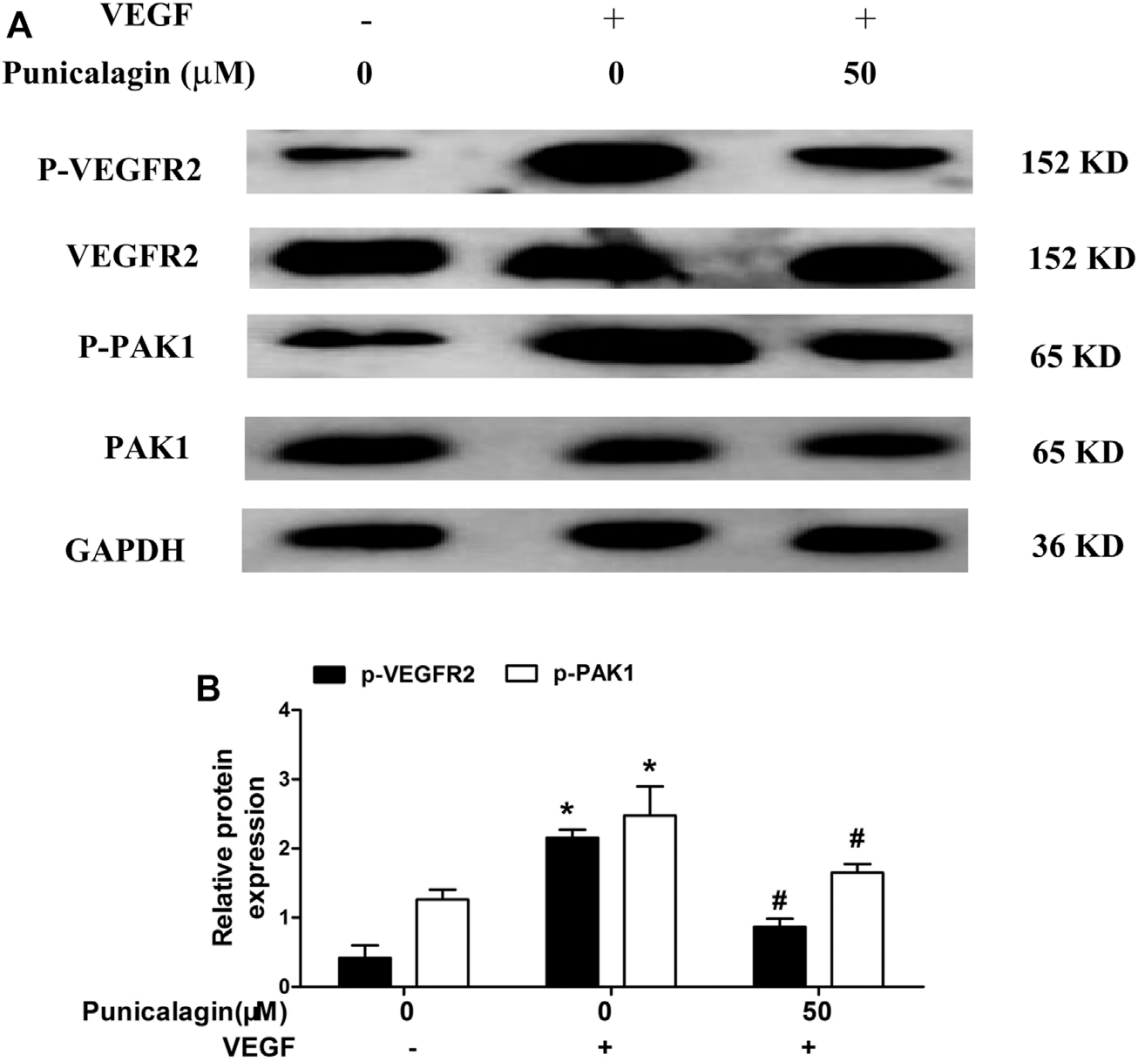
Inhibitory effect of punicalagin on VEGF-induced phosphorylation of VEGFR2 and PAK1. **(A)** Protein expression levels of phospho-VEGFR2 and phospho-PAK1 normalized by GAPDH. **(B)** The densitometric analysis of phospho-VEGFR2 and phospho-PAK1 *p < 0.05, VEGF treatment versus control group, ^#^ *p* < 0.05 TNF-α + punicalagin treatment vs. treatment with VEGF alone.

To further elucidate the anti-angiogenesis mechanism of punicalagin, we performed western blot to detect the phosphorylation of VEGFR2 and PAK1. As shown in Figures 10A,B **,** punicalagin could significantly reduce the expression of phosphorylated protein caused by VEGF stimulation (*p* < 0.05).

### Effect of Punicalagin on Leukocyte Infiltration in Mice Model of Acute Lung Injury

**FIGURE 11:**
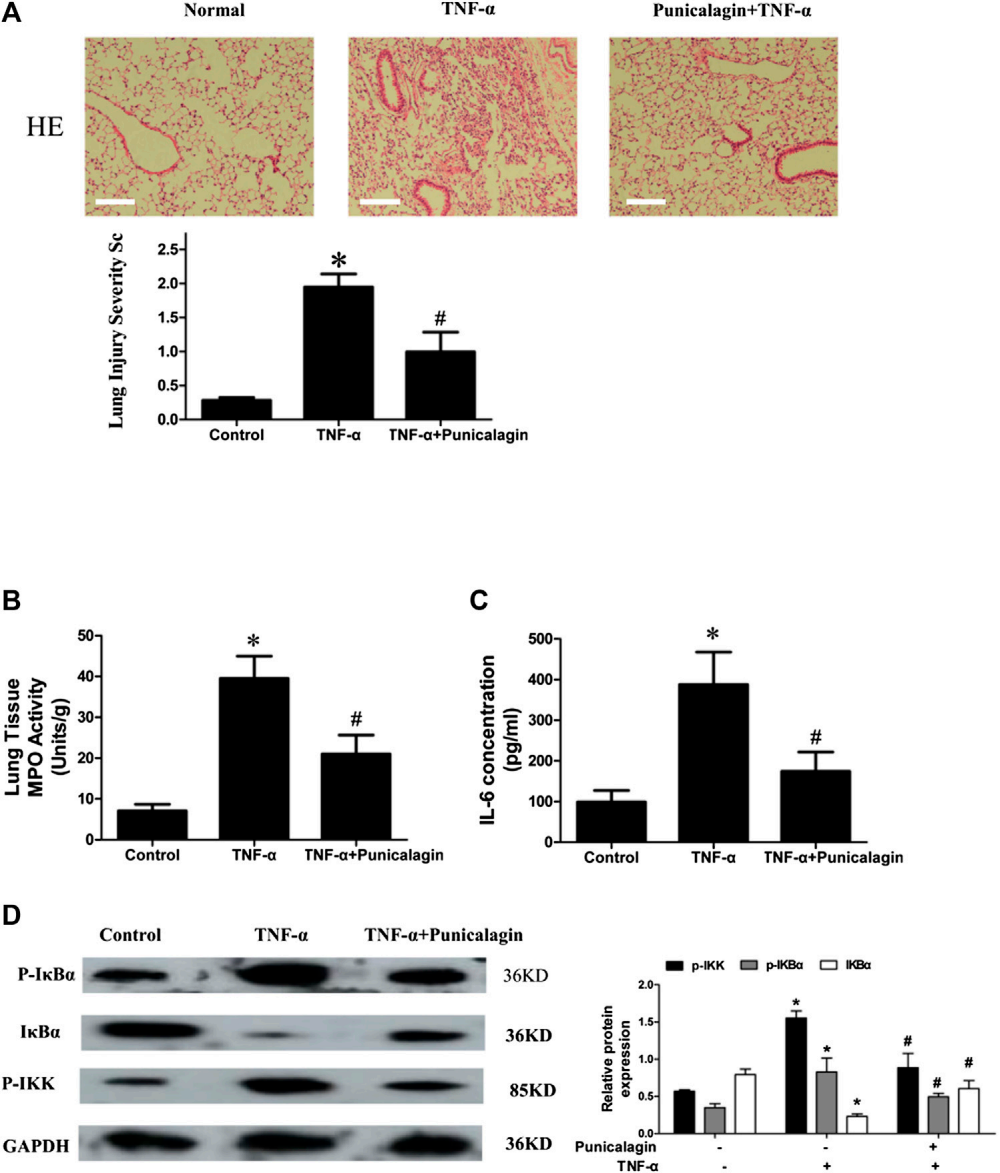
*In vivo* model and inhibitory effect of punicalagin on neutrophil infiltration. **(A)** H&E staining to assess leukocyte accumulation in the lung and to quantify the severity of injury. Magnification: 200× **(B, C)** MPO activity measured by erythroperoxidase assay kit and IL-6 secretion detected with ELISA. Punicalagin inhibited TNF-α-induced activation of NF-κB with western blotting **(D)**. Data are represented as mean ± SD. *p < 0.05, punicalagin treatment vs control group. ^#^ *p* < 0.05 TNF-α+punicalagin treatment vs. treatment with TNF-α alone.

Acute lung injury (ALI) is an inflammatory lung disease which is characterized by infiltrated in the lung tissue with great mount of inflammatory mediator and inflammatory cells (Liu et al., 2016). Our above research showed that punicalagin plays a vital role in anti-inflammatory and anti-angiogenesis *in vitro*. To confirm the effect of punicalagin on inflammation *in vivo*, we established a mouse model of acute lung injury. We investigated the effects of punicalagin by pathological staining and detected MPO activity and inflammatory factors. HE staining showed that after 5 h of treatment with TNF-α alone, numerous neutrophils infiltrated the mouse lung tissue (Figure 11A). Myeloperoxidase (MPO) is the most abundant enzyme in neutrophils. It can kill microorganisms and destroy a variety of targeted substances in phagocytes, play a role in regulating inflammation in the body. As a multifunctional inflammatory factor, IL-6 has multiple biological functions and is the main cytokine that mediates acute lung injury. Pre-treatment with punicalagin significantly suppressed the production of MPO in neutrophils and levels of TNF-α-induced inflammatory cytokine IL-6 (Figures 11B,C) (*p* < 0.05). Western blot analysis showed that TNF-α challenge markedly increased the phosphorlation of IKKβ, IκBα and degradation of IκBα.Whereas, pretreatment with punicalagin inhibited the phosphorlation of IKKβ, IκBα, and the degradation of IκB-α (Figure 11D).

## Discussion

Endothelial cells (ECs) are fundamental components of blood vessels. The present findings demonstrated that punicalagin could inhibit endothelial inflammatory responses of HUVECs induced by TNF-α via the decrease in the expression of pro-inflammatory cytokines (IL-6, IL-8 and MCP-1), as well as adhesion molecules (ICAM-1 and VCAM-1). Besides, punicalagin suppressed the migration, tube formation, and adhesion of mononuclear leukocytes to endothelial cells *in vitro*. Furthermore, punicalagin could decrease the IKK-mediated activation of NF-κB pathway in TNF-α-induced ECs and suppressed the VEGF-induced endothelial VEGFR2 and PAK1 activation. *In vivo* studies, we found that punicalagin pretreatment could alleviate septic response in acute lung injury mouse, as compared with the normal control. Taken together, these findings revealed that punicalagin could serve as a novel drug for vascular inflammation.

As a major inflammatory factor, TNF-α has been reported to mediate the interaction between endothelial cells and effector monocytes (Liu and Tie, 2019). In addition, TNF-α also plays a vital role in atherosclerotic inflammatory progression through raise expression of adhesion molecule and activation of ECs (Zhang et al., 2020). Adhesion molecules (e.g. VCAM-1 and ICAM-1) have been shown to have pathogenic functions in inflammatory and atherosclerotic processes (Nakashima et al., 1998). HUVECs are critical for maintaining vascular homeostasis, such as regulation of leukocyte transport, vascular permeability, and blood fluidity, in the early stages of normal inflammatory responses and related diseases (Berlin et al., 1995; Kansas, 1996). In our study, qRT-PCR analysis suggested that the suppressive effect of punicalagin on expression of ECs adhesion molecules and cytokines, which was correlated with their mRNA levels. Moreover, punicalagin attenuated the attachment of mononuclear leukocytes to ECs. These findings demonstrated that punicalagin play a vital role in attenuating vascular inflammation at the early stages. There are studies revealed that the induction of ICAM-1, VCAM-1 and other cytokines in ECs is primarily modulated via NF-κB signaling pathways (Hou et al., 1994; Manning et al., 1995). Once ECs was stimulated by TNF-α, P65 translocated from cytoplasm to the nucleus and NF-κB activated (Nežić et al., 2018). It was reported that chronic neuroinflammation were ameliorated by punicalagin via NF-κB inhibition in neuronal cells (Kim et al., 2017). Consistent with the inhibitory effect on neuro-inflammation, the results of our study revealed that punicalagin have remarkably effect on inhibition of the TNF-α-induced phosphorylation, disintegration of IκBα and translocation of nuclear NF-κB in HUVECs. The inhibitory effect of punicalagin on TNF-α induced NF-κB activation further support the notion that punicalagin might play a critical role in inhibiting vascular inflammation.

HUVECs barrier integrity is critical to the functions of tissues and organs. The increase in endothelial permeability respond to inflammatory mediators and cytokines could cause local edema. Persistently, unsolved endothelial hyperpermeability might underly the initiation and progression of chronic inflammatory diseases, including atherosclerosis and rheumatoid arthritis (Atehortúa et al., 2019). Therefore, the conservation of vascular HUVECs barrier integrity might have great potential for clinical application. In the present study, it was found that punicalagin could suppress VEGF165-induced endothelial hyperpermeability in TEER assay. Furthermore, treatment with punicalagin suppressed leukocyte infiltration via permeable endothelium in TNF-α-induced acute inflammation in murine lungs. This indicated that endothelial punicalagin prevents leukocyte infiltration in acute inflammation through, at least in part, decreasing vascular hyperpermeability.

Angiogenesis is the expansion and remodeling of blood vessels. It is a multistep and intricate cascade involving migration, proliferation and organization into capillary tubular structures of ECs. Moreover, angiogenesis plays a crucial role in normal physiological processes (e. g. embryonic development and wound healing), as well as in pathological states, including but not least cancer, diabetes, cardiovascular disease, and inflammatory diseases. Further, modulation of angiogenesis has been considered as a promising approach for treating cancer. It has been reported that punicalagin had a positive effect of inhibit tumor angiogenesis (Huang et al., 2020). In agreement with our research, punicalagin suppressed VEGF165-induced tube formation *in vitro*, suggesting an important role in controlling angiogenesis. Migration of endothelial cells in response to angiogenic growth factors (e. g., VEGF) is crucial to this process. VEGF is also important in physiological and pathological angiogenesis. VEGF could react with receptor tyrosine kinases including VEGFR1 and VEGFR2 (Melincovici et al., 2018). VEGFR2 is a key transducer of VEGF signal in ECs. It can stimulate cell proliferation, migration, differentiation, capillary-like structure formation, and enhance blood vessel permeability (Hou et al., 1994; Shibuya, 2013). PAK1 has been proved to regulate endothelial cell proliferation, migration and angiogenesis (Huang et al., 2016). PAK1 depletion could lead to severe apoptosis, increased vascular permeability, and angiogenesis defect in mice (Radu et al., 2015). Thus, our results demonstrated the inhibitory effects of punicalagin on VEGF165-induced activation of VEGFR2 and PAK1 in HUVECs.

In summary, we have identified punicalagin as a drug that inhibits endothelial permeability, inflammation and angiogenesis via regulating NF-κB and VEGFR2/PAK1 signaling pathways. Our study supports the hypothesis that punicalagin may become an essential pharmacological agent by controlling key aspects of HUVECs homeostasis in physiologic variation or pathologic states. The anti-inflammatory and anti-angiogenic effect of punicalagin has critical implications for its clinical application as a preventative or treatment option for rheumatoid arthritis, and cardiovascular disease, inflammatory diseases and cancer.

## Data Availability

The raw data supporting the conclusions of this article will be made available by the authors, without undue reservation.
