# Supplementary material for: Inhibitory Effect of Punicalagin on Inflammatory and Angiogenic Activation of Human Umbilical Vein Endothelial Cells
Source: Front Pharmacol. 2021 Nov 16;12:727920. doi: 10.3389/fphar.2021.727920 (PMC8636678; doi:10.3389/fphar.2021.727920)
Supplement: Supplementary file 1 [file Table1.pdf]

STable 1 The sequences of RT-PCR primers

	sequences	
IL-6	Forward	ACTCACCTCTTCAGAACGAATTG
	Reverse	CCATCTTTGGAAGGTTCAGGTTG
IL-8	Forward	ACTGAGAGTGATTGAGAGTGGAC
	Reverse	AACCCTCTGCACCCAGTTTTC
MCP-1	Forward	CAGCCAGATGCAATCAATGCC
	Reverse	TGGAATCCTGAACCCACTTCT
VCAM-1	Forward	CAGTAAGGCAGGCTGTAAAAGA
	Reverse	TGGAGCTGGTAGACCCTCG
ICAM-1	Forward	GTATGAACTGAGCAATGTGCAAG
	Reverse	GTTCCACCCGTTCTGGAGTC
GAPDH	Forward	GCACCGTCAAGGCTGAGAAC
	Reverse	TGGTGAAGACGCCAGTGGA
